# MEASURING OLDER ADULT LONELINESS ACROSS COUNTRIES

**DOI:** 10.1093/geroni/igac059.1172

**Published:** 2022-12-20

**Authors:** Lauren Newmyer, Ashton Verdery, Rachel Margolis, Lea Pessin

**Affiliations:** The Pennsylvania State University, University Park, Pennsylvania, United States; The Pennsylvania State University, University Park, Pennsylvania, United States; Western University, London, Ontario, Canada; The Pennsylvania State University, University Park, Pennsylvania, United States

## Abstract

The topic of older adult loneliness commands increasing media and policy attention around the world. Are surveys of aging equipped to measure it? We assess the measurement of loneliness in large-scale aging studies in 31 countries. In each country, we document available loneliness measures, examine correlations between different measures, and assess how these correlations differ by gender and age group. There is substantial heterogeneity in available measures of loneliness across countries. Within countries with multiple measures, the correlations between measures are high (range .38-.78). Differences by age and gender group are relatively small. Correlations between loneliness measures and living alone and being without a spouse are positive and similar in magnitude across countries, supporting construct validity. We establish that even single-item measures of loneliness contribute meaningful information in diverse contexts, with reliable and consistent measurement properties within many countries.

